# Self‐control and grit are associated with school performance mainly because of shared genetic effects

**DOI:** 10.1002/jcv2.12159

**Published:** 2023-04-26

**Authors:** Sofieke T. Kevenaar, Conor V. Dolan, Dorret I. Boomsma, Elsje van Bergen

**Affiliations:** ^1^ Department of Biological Psychology Vrije Universiteit Amsterdam Amsterdam The Netherlands; ^2^ Research Institute LEARN! Vrije Universiteit Amsterdam Amsterdam The Netherlands; ^3^ Amsterdam Public Health Research Institute Amsterdam The Netherlands; ^4^ Amsterdam Reproduction and Development Research Institute Amsterdam The Netherlands

**Keywords:** academic achievement, grit, heritability, non‐cognitive skills, school performance, self‐control

## Abstract

**Background:**

By combining the classical twin design with regression analysis, we investigated the role of two non‐cognitive factors, self‐control and grit, in the prediction of school performance. We did so at the phenotypic, genetic, and environmental level.

**Methods:**

Teachers filled out a survey on the twins' school performance (school grades for reading, literacy, and math), self‐control (ASEBA self‐control scale), and grit (the perseverance aspect) for 4891 Dutch 12‐years‐old twin pairs (3837 pairs with data for both and 1054 pairs with data for one of the twins). We employed regression analyses to first assess the contributions of self‐control and grit to school performance at the phenotypic level, and next at the genetic and environmental level, while correcting for rater (teacher) effects, parental SES, and sex.

**Results:**

Higher SES was associated with better school performance, self‐control, and grit. On average, girls had more self‐control and grit than boys. Corrected for sex, SES, and teacher rater effects, genetic factors accounted for 74%, 69%, and 58% of the phenotypic variance of school performance, self‐control, and grit, respectively. Phenotypically, self‐control and grit explained 28.3% of the variance in school performance. We found that this phenotypic result largely reflected genetic influences.

**Conclusions:**

Children who have better self‐control and are grittier tend to do better in school. Individual differences in these three traits are not correlated because of shared environmental influences, but mainly because of shared genetic factors.

## Graphical abstract

This study used a genetically‐informed regression (on *N* = 4891 12‐years‐old twin pairs) to investigate the relationship between self‐control, grit, and school performance. The results indicate that genetic factors play a significant role in explaining the association between these non‐cognitive factors and school performance. Specifically, children who exhibit greater self‐control and grit tend to perform better in school, and this relationship is primarily due to shared genetic factors rather than shared environmental influences.

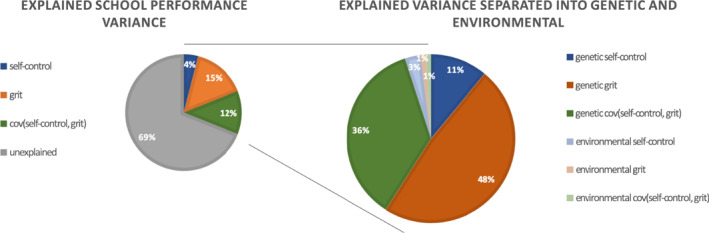

**Key points**

- Cognitive factors are known to influence school performance. Less is known about the contribution of non‐cognitive factors.
- In 3837 complete and 1054 incomplete twin pairs, we found that non‐cognitive skills, especially grit, predict school performance.
- The non‐cognitive skills self‐control and grit explain ∼28% of individual differences in school performance at the phenotypic level.
- Both school performance and non‐cognitive skills are heritable (58%–74%).
- The regression relationship between school performance and non‐cognitive factors mainly reflects genetic influences.
- Future work could investigate whether interventions targeting non‐cognitive skills improve school performance.

## INTRODUCTION

Understanding individual differences in school performance is important given the large influence they have across all domains of life. Cognitive variables, such as intelligence, are important for school success, but these only explain part of the individual differences (Bartels et al., 2002; Kautz et al., 2014). Here, we considered the role of non‐cognitive factors. Two such factors that have been related to school success are self‐control and grit. Self‐control is defined as the “capacity to resist temptation or inhibit a dominant response or activate a subdominant response” (Nigg, 2017, p. 364). Grit is defined as perseverance and passion for long‐term goals (Duckworth et al., 2007). Grit has two aspects: consistency of interest and perseverance of effort (Duckworth et al., 2007). Of these, perseverance of effort is more strongly linked to school performance (Credé, Tynan, & Harms., 2017; Muenks et al., 2017; Rimfeld et al., 2016). The grit measure that we analyzed in this study mostly relates to this perseverance of effort aspect, especially as manifest in the classroom setting. We investigated the differential prediction by self‐control and grit of individual differences in school performance of 12‐year‐olds in whom we collected data on these measures from their schoolteachers in a prospective study design. We analyzed the relationship between school performance and non‐cognitive factors both at the phenotypic level and at the genetic and environmental levels.

Self‐control and grit are distinct, but strongly correlated concepts (*r* ∼ 0.60; Duckworth & Gross, 2014; Duckworth et al., 2007). Grit entails persistent focused effort and long‐term commitment to goals, whereas self‐control encompasses the capacity to regulate attention, emotion, and behavior in the presence of distractions and temptations (Duckworth et al., 2007; Duckworth & Gross, 2014). Self‐control keeps one focused on a task at hand and is required in (and outside) the school context. Grit involves making appropriate choices to reach a long‐term goal. So, grit is needed to persevere in working toward a higher‐order long‐term goal, while self‐control is needed to resist short‐term distractions and temptations (Duckworth & Gross, 2014). Empirical studies have demonstrated the associations between self‐control, grit, and other non‐cognitive factors, like the Big Five personality trait conscientiousness (Credé et al., 2017; Muenks et al., 2017). Conscientiousness can be defined as being “self‐disciplined, responsible, hardworking and thorough” (John & Srivastava, 1999). Werner et al. (2019) showed that self‐control, grit, and conscientiousness explained 10% of the variance in academic motivation.

Multiple studies have documented that school performance is correlated with self‐control, grit, and conscientiousness. These three non‐cognitive skills are overlapping constructs and poorly distinguishable (Muenks et al., 2017; Ponnock et al., 2020; Takahashi et al., 2021). Duckworth et al. (2014, 2019) showed that performance on standardized achievement tests administered at school was predicted by non‐cognitive skills like self‐control, motivation, and study strategies, in addition to socioeconomic status and general intelligence. Oriol et al. (2017) showed in primary school children that grit is related to academic self‐efficacy, while self‐control is related to school satisfaction. Usher et al. (2019) found that grit correlated modestly with self‐efficacy (*r* ∼ 0.50), but weaker with teacher ratings in reading and math (*r* ∼ 0.20), and with achievement test scores (*r* ∼ 0.10). Self‐efficacy was weakly to moderately related to all outcomes (*r* ∼ 0.30). Of note is that a meta‐analysis confirmed that of the two facets of grit, perseverance of effort and consistency of interest, the perseverance facet is much more strongly related to academic performance (*ρ* = .26) than the consistency facet (*ρ* = .10; Credé et al., 2017). We therefore focus on perseverance.

Cognitive skills, school performance, and education‐related traits are heritable, with genetic differences being the main source of individual differences. That is, for most societies that have been included in behavior genetic studies. In 12‐year‐old children in The Netherlands, the estimated heritability (i.e., the proportion of variance attributable to genetic influences) of standardized‐test performance at the end of primary school is 74%. That is, 74% of test‐score differences among children are due to genetic differences. Only 8% of individual differences were accounted for by shared‐environmental influences (de Zeeuw et al., 2016). Shared‐environmental influences common to children growing up in the same family contribute to the resemblance of twins and siblings. A recent meta‐analysis of twin studies found self‐control to be 60% heritable (Willems et al., 2019). Interestingly, the 40% environmental effects on self‐control were not shared by twins. This may imply that the environmental effects do not originate in aspects of the rearing environment that are likely to be shared, such as parental upbringing or parental style, but experiences unique to each sibling, stemming from, for instance, illness, different friends, and stochastic influences (Tikhodeyev & Shcherbakova, 2019; Willems et al., 2019). The heritability of grit has been estimated at 35%–61%, and like self‐control, grit shows no evidence of shared environmental effects (Martinez et al., 2022; Rimfeld et al., 2016; Tucker‐Drob et al., 2016). Martinez et al. (2022) investigated grit and mindset in relation to reading comprehension in 422 thirteen‐ and fifteen‐year‐old twin pairs. Individuals can hold the belief that intelligence is mainly a fixed inborn trait (fixed mindset) or a malleable trait given effort and time (growth mindset). Grit and mindset were correlated with reading ability, but mindset and grit were not associated with the change in reading ability over time (Martinez et al., 2022). In a review, Malanchini et al. (2020) concluded that non‐cognitive abilities explained genetic variance in academic performance above and beyond cognitive ability. The strong stability and heritability of academic performance appear to be partially driven by additional factors besides cognitive ability.

In conclusion, school performance, self‐control, and grit are related traits that are subject to genetic influences, and self‐control and grit predict school performance. Here we set out to determine the degree to which genetic and environmental factors contribute to the phenotypic relationships between these non‐cognitive factors and school performance. We addressed this question by applying simultaneous regression and genetic covariance modeling, as outlined in Boomsma et al. (2021).

Analyzing twin data, we can distinguish genetic and environmental sources of variation. We analyzed data that were collected from the teachers of children. Teachers assessed school performance across three domains and assessed self‐control and grit. This feature of the data poses a challenge: twins in the same class were rated by the same teacher, while twins in different classes (or schools) were rated by different teachers. As teachers may have their unique views of children, and their style of rating them, in our models we included random teacher‐rater effects. In so doing, we distinguished variance due to raters and variance due to child factors. We included sex and parental SES as covariates (Gil‐Hernández, 2021). So, we accounted for the random effect of rater and the fixed effects of sex and parental SES.

## METHODS

### Participants

We included data from 11.5 to 12.5‐year‐old twins registered in the young Netherlands Twin Register (NTR). The young NTR includes twins and multiples born in 1986, and their parents, siblings, and teachers, who participate in longitudinal research (Boomsma et al., 2006). More information about data collection, recruitment, and response rates can be found elsewhere (Ligthart et al., 2019; Van Beijsterveldt et al., 2013). Young twins are registered by their parents, usually a few weeks to months after birth. For the data used in this paper, the parents are approached when the twins are 12 years old with a request for permission to approach their teachers for ratings of behavior in school and school performance. Parents, who grant permission, then provide the name of the teacher and the address of the school. Teachers are subsequently invited to complete a survey concerning the twin(s) in their class.

Our sample included 3837 pairs with data on both twins and 1054 incomplete pairs, that is, pairs with data on one twin member. These incomplete pairs arose because some of the twins were in different classes and rated by different teachers, so the teacher of one twin might have completed the survey, but the teacher of the other did not. There were 1957 monozygotic and 2934 dizygotic twin pairs with school performance, self‐control, and/or grit measures. The zygosity of the same‐sex twin pairs was determined by a DNA test (32.2% of the same‐sex pairs) or by a questionnaire with items concerning the twin resemblance, which the parents completed. Based on this questionnaire, zygosity is correctly determined in over 95% of the cases (Ligthart et al., 2019). The data collection procedure was ethically approved by the Vaste Commissie Wetenschap en Ethiek at Vrije Universiteit Amsterdam (VCWE‐2021‐111).

### Measures

#### Self‐control

Self‐control was assessed by the Achenbach Self‐Control Scale (ASCS; Willems et al., 2018) in the ASEBA‐TRF reported by teachers. The scale consists of eight items, displayed in Table 1, scored on a 3‐point response scale. The response options are 0 (*not true*), 1 (*somewhat or sometimes true*), and 2 (*very true or often true*). Cronbach's α of the ASCS is 0.82 for teacher reports at age 12. The internal inter‐rater and test‐retest reliability are good (Willems et al., 2018). If three or fewer items were missing (34.5% of the sample due to ASEBA‐TRF version changes over the years), the mean of the available items was substituted for the missing items to compute the sum score, as described by Willems et al. (2018). If more than three items were missing the sum scores was coded as missing. We reverse‐coded the item scores so that a higher score indicated greater self‐control. The total score ranged from 0 to 16.

**TABLE 1:** The Achenbach Self‐Control Scale items to assess self‐control problems and the items to assess grit.

Self‐control items	Grit items
Fails to finish things he/she starts	Compared to typical pupils of the same age:
Can't concentrate, can't pay attention for long	‐ How hard does he/she work?
Breaks rules at home, school or elsewhere	‐ How appropriately does he/she behave?
Impulsive or acts without thinking	‐ How task‐oriented is he/she?
Inattentive or easily distracted	
Stubborn, sullen or irritable	
Sudden changes in mood or feelings	
Temper tantrums or hot temper	

*Note*: For self‐control we reversed the scores (higher scores indicate more self‐control). Self‐control items were scores on a 3‐point scale and grit items on a 7‐point scale.

#### Grit

The grit measure was based on teacher reports on two or three items, namely *Compared to typical pupils of the same age, 1) how hard does he/she work*; *2) how appropriately does he/she behave*, and *3) how task‐oriented is he/she*. The response format was a 7‐point Likert scale. Due to changes in YNTR surveys over the years, the third item was missing in 55.2%. The item scores were summed to sum scores. If more than one item was missing, the grit score was coded as missing. If a single item was missing (mostly item 3), the mean of the other two items was imputed for the missing item. The sum scores range from 1 to 21, with higher scores indicating more grit. The correlations among the grit items are 0.70 (items 1 and 2), 0.71 (items 2 and 3) and.82 (items 1 and 3). The correlation between the grit measure as used in the paper and the two most relevant items for grit is high (0.88; see Table 2) and justifies our use of the measure. Cronbach's α is 0.87.

**TABLE 2:** Correlations among school performance, self‐control, grit, CITO, and IQ.

	Self‐control	Grit	Grit items 1 & 3	School performance	Reading	Literacy	Math	CITO	IQ
Self‐control		0.66 (8459)	0.56 (3405)	0.40 (8087)	0.29 (6240)	0.36 (7555)	0.33 (7936)	0.29 (4642)	0.28 (417)
Grit			0.88 (3420)	0.53 (8090)	0.38 (6245)	0.46 (7564)	0.44 (7937)	0.42 (4625)	0.32 (416)
Grit items 1 & 3				0.48 (3393)	0.40 (3440)	0.49 (3437)	0.46 (3436)	0.43 (1061)	N.A. (0)
School performance					0.79 (6257)	0.85 (7569)	0.80 (7945)	0.70 (4381)	0.51 (377)
Reading						0.73 (6137)	0.51 (6302)	0.57 (3031)	0.43 (180)
Literacy							0.67 (7607)	0.67 (4115)	0.47 (325)
Math								0.72 (4242)	0.58 (355)
CITO									0.67 (331)
*N*	8521	8490	3496	8128	6401	7740	8130	4723	421
Mean	14.09	14.87	10.20	11.50	3.88	3.81	3.83	538.13	100.16
SD	2.66	4.09	2.82	2.98	1.13	1.06	1.19	8.48	13.50

*Note*: This table is based on the raw data, uncorrected for sex, SES, teacher sharing, and censoring. The numbers between brackets refer to the number of children with overlapping data of the two constructs. Grit items 1 and 3 include the items “How hard does he/she work” and “how task‐oriented is he/she?” (so leaving out item 2). Reading, Literacy and Math are the teacher‐reported school grades, and school performance is the sum of these grades. CITO is the score on the nationally‐standardized school test at the end of primary school (Grade 6, ∼12yo). IQ = score on the WISC‐R.

All correlations are significant at *p* < .001.

#### School performance (grades and CITO standardized test)

Teachers reported the grades for math, reading, and literacy on 5‐point scales, with scale points 1 (*fail*), 2 (*poor*), 3 (*satisfactory*), 4 (*above average*), and 5 (*good or excellent*) (de Zeeuw et al., 2014; van Bergen et al., 2018). The responses to these three items were summed, as detailed in de Zeeuw et al. (2016). School performance scores ranged from 3 to 15, with higher scores indicating better performance. If a single rating was missing (22.9% of the cases), the mean of the other two ratings was substituted. If more than one item was missing, the school performance score was coded as missing. Reading grades correlated 0.73 with literacy grades and 0.51 with math grades. Literacy grades and math grades correlated 0.67 (see Table 2).

In about half of the twins (*N* = 4723 individual children), we had scores on a nationwide standardized educational‐achievement test (i.e., CITO scores; Centraal Instituut voor Toets Ontwikkeling, 2002; de Zeeuw et al., 2020), to validate teacher‐reported school performance. The CITO is a high‐stakes test at the end of primary school (Grade 6; ages 11 or 12) that is taken at school over three mornings. CITO scores correlated highly with the reported school grades and with the sum score, our measure of school performance (Table 2). Both teacher reports and test scores are heritable, reliable, and predictive of future academic achievement (Rimfeld et al., 2019; van Bergen et al., 2018). When we refer to school performance in this paper, we refer to the sum of the teacher‐reported school grades, because this measure was available for most children and overall and the correlation with the standardized CITO test was high (0.70; see Table 2).

#### Sex and socioeconomic status (SES)

Sex was coded 1 for males and 2 for females. SES was based on a combination of parental occupation and parental education (for details, see de Zeeuw et al., 2019), and was coded 1 (*lowest SES*) through 4 (*highest SES*).

#### Teacher sharing

Twins may or may not be in the same class. Twins in the same class were rated by the same teacher, while twins in different classes were rated by different teachers. This sometimes resulted in incomplete pairs, where one teacher participated in the study and the other did not. Teacher sharing was coded 1 (twins in the same class, rated by the same teacher) or 0 (different classes, different teachers).

#### IQ

A subsample of 421 children was assessed on full‐scale IQ, by the full Dutch WISC‐R (van Haasen et al., 1986). There are 12 subscales, of which half focus on verbal and the other half focus on non‐verbal IQ. For a detailed description of these data, see the age‐12 assessment in Bartels et al. (2002). The IQ measure in the subsample allowed us to test if our non‐cognitive skills predict school performance over and above IQ.

### Statistical analyses

We started with testing, in the IQ subsample, whether our non‐cognitive factors explain variance in school performance over and above the cognitive factor IQ. Then we moved on to our main analyses.

To assess the differential relationship of self‐control and grit with school performance, we first carried out phenotypic regression analysis, followed by genetic and environmental regression analyses (Boomsma et al., 2021) to determine the contributions of self‐control and grit, and their covariance, to the variance in school performance at the phenotypic, genetic, and environmental level. The data were negatively skewed because of a ceiling effect, hence we corrected for censoring in all analyses (see de Zeeuw et al., 2019). We fitted the models using full information maximum likelihood estimation, assuming that the data follow a censored multivariate normal distribution.

First, we carried out phenotypic regression analyses, in which we regressed school performance (SP) on self‐control (SC) and grit, and on the covariates sex, SES, and teacher sharing (*t*, coded 0/1).

$${SP}_{i}=b_{0}+b_{sex}*{sex}_{i}+b_{SES}*{SES}_{i}+t_{i}+b_{SC}*{SC}_{i}+b_{grit}*{grit}_{i}+\varepsilon_{i}$$

with subscript *i* representing individual, *b*_0_ representing the intercept, and *ε*_i_ representing prediction error. The term *t*_i_ is the random teacher effect. Conditional on sex, SES, and teacher sharing, the phenotypic school performance variance was decomposed into four parts:

$$\Sigma_{SP|sex,SES,teacher}^{2}=b_{SC}^{2}*\Sigma_{SC}^{2}+b_{grit}^{2}*\Sigma_{grit}^{2}+\left(2*b_{SC}*b_{grit}*\Sigma_{SC,grit}\right)+\Sigma_{\varepsilon }^{2}$$

**FIGURE 1:**
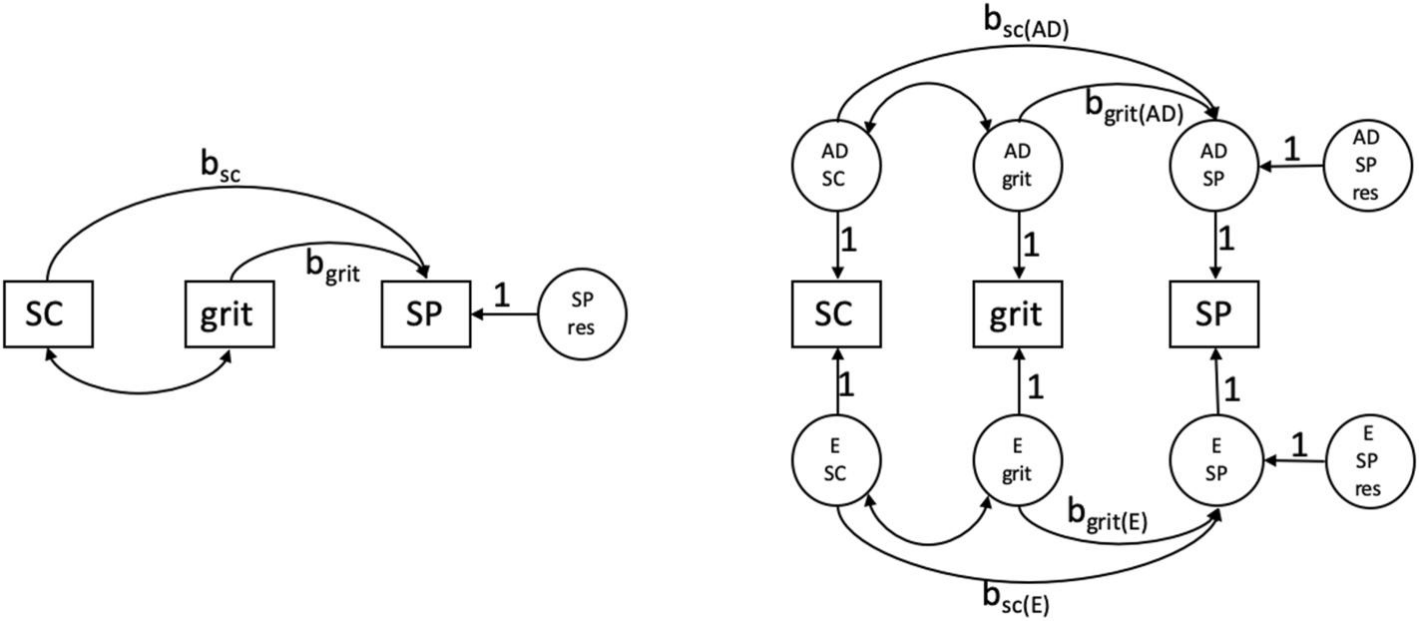
Figure on the left side: path diagram of the phenotypic regression model of school performance on self‐control and grit, conditional on sex and SES and teacher (omitted in the figure). b_SC_ and b_grit_ represent the regression coefficients of self‐control and grit respectively. Figure on the right side: path diagram of the regression of school performance on self‐control and grit including the genetic (AD) and environmental (E) latent factors. The parameters b_SC(AD)_, b_grit(AD),_ b_SC(E),_ and b_grit(E)_ represent regression coefficients. AD SP res represents the residual genetic term of school performance and E SP res represents the residual environmental term of school performance.

The term 2 * *b*_SC_ * *b*_grit_ * Σ_SC,grit_, due to the covariance of self‐control and grit (Σ_SC,grit_), captures variance that cannot be unambiguously attributed to either self‐control or grit. We fitted the same regression model simultaneously to the data of all MZ and DZ twins, taking into account that the scores within twin pairs are dependent. The left side of Figure 1 displays the phenotypic model.

**FIGURE 2:**
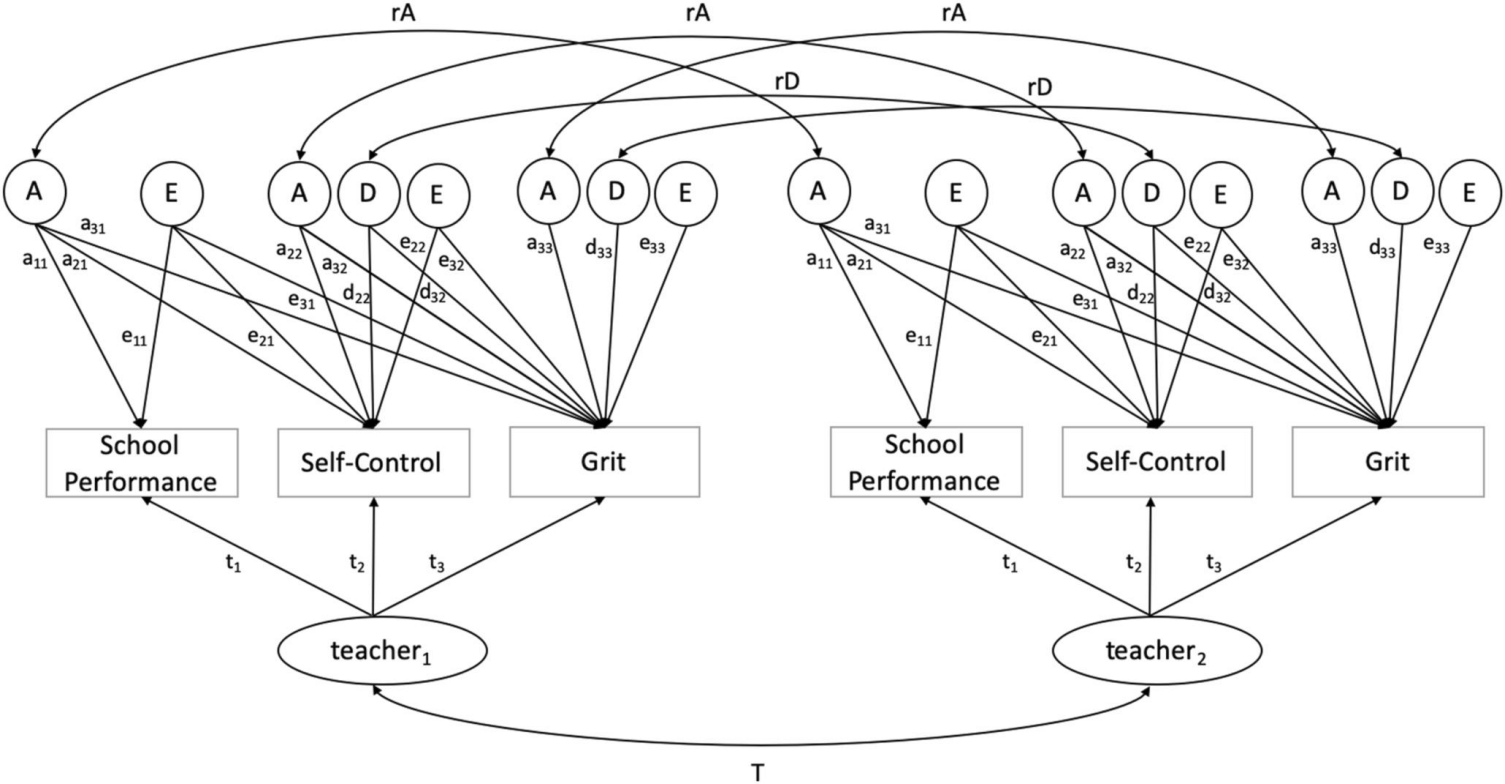
Path diagram of the genetical structural equation model, with twin one of a pair displayed on the left side of the figure and twin two of the same pair displayed on the right. rA denotes the correlation between the A factors in twin 1 and twin 2 and is fixed to one in MZ twins and to 0.5 in DZ twins. rD denotes the correlation between the D factors in twin 1 and twin 2 and is fixed to one in MZ twins and to 0.25 in DZ twins. T denotes teacher sharing and is fixed to 1 for twins who share a teacher and 0 for twins who do not share a teacher. The covariates SES and sex are omitted from this figure.

**FIGURE 3:**
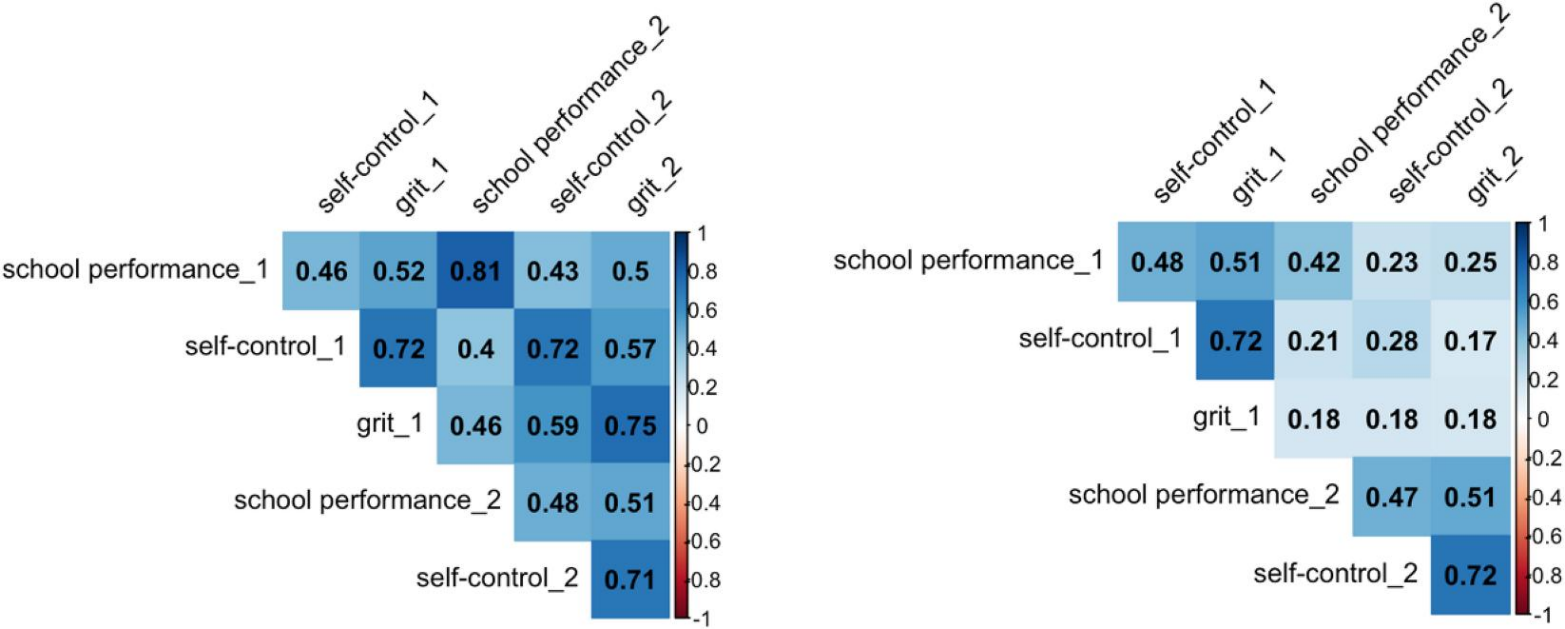
Twin correlations for MZ (left) and DZ (right) twins corrected for sex, SES, teacher sharing and censoring. First‐born twins of a pair are indicated with “_1” and second‐born twins with “_2”. The figure includes cross‐twin within‐trait correlations (= the correlation between twin 1 and twin 2 for the same trait), cross‐twin cross‐trait correlations (= the correlation between twin 1 and twin 2 for the different traits) and within‐twin cross‐trait correlations (= the correlation between different traits in the same twin).

Next, we fitted a genetic structural equation model (Figure 2). In earlier research, self‐control and grit were found to be influenced by additive genetic effects and genetic dominance effects. Finding genetic dominance implies non‐additive genetic effects of certain alleles (for an in‐depth explanation, see Falconer & Mackay, 1983). By the common rule of thumb, we infer dominance if *r*_MZ_ > 2*r_DZ_, where r_MZ_ and r_DZ_ are the MZ and DZ twin correlations. Given the twin correlations in Figure 3, we fitted a model including additive genetic effects (A), dominance effects (D), and unshared environmental effects (E). As shown below, we calculated the MZ and DZ covariance matrices based on the estimated additive genetic Σ_A_ and the dominance Σ_D_ covariance matrices (the dominance effects limited to self‐control and grit), and the unshared environmental Σ_E_ covariance matrix. We included the covariance matrix Σ_T_ to accommodate possible rater (teacher) variance (see below). The 3 × 3 additive genetic covariance matrix Σ_A_, the 3 × 3 covariance matrix Σ_D_, and the 3 × 3 unshared environmental covariance matrix Σ_E_ were modeled using triangular decomposition, as *Σ*_A_ = Λ_A_Λ_A_^t^, Λ_D_Λ_D_^t^ and *Σ*_E_ = Λ_E_Λ_E_^t^, respectively, where

$$\Lambda_{A}=\begin{array}{cc} \begin{array}{c} School\;performance\\ Self-control\\ Grit \end{array} & \begin{array}{c} \;\mathrm{SP}\;\mathrm{SC}\;\mathrm{Grit}\\ \left[\begin{array}{ccc} a_{11} & 0 & 0\\ a_{21} & a_{22} & 0\\ a_{31} & a_{32} & a_{33} \end{array}\right] \end{array} \end{array}$$

$$\Lambda_{D}=\begin{array}{cc} \begin{array}{c} School\;performance\\ Self-control\\ Grit \end{array} & \begin{array}{c} \;\mathrm{SP}\;\mathrm{SC}\;\mathrm{Grit}\\ \left[\begin{array}{ccc} d_{11} & 0 & 0\\ d_{21} & d_{22} & 0\\ d_{31} & d_{32} & d_{33} \end{array}\right] \end{array} \end{array}$$

and

$$\Lambda_{E}=\begin{array}{cc} \begin{array}{c} School\;performance\\ Self-control\\ Grit \end{array} & \begin{array}{c} \;\mathrm{SP}\;\mathrm{SC}\;\mathrm{Grit}\\ \left[\begin{array}{ccc} e_{11} & 0 & 0\\ e_{21} & e_{22} & 0\\ e_{31} & e_{32} & e_{33} \end{array}\right] \end{array} \end{array}$$

Lastly, the random teacher‐rater effect was modeled using the 3 × 3 covariance matrix Σ_T_, which was modeled as Σ_T_ = Λ_T_Λ_T_^t^, where

$$\Lambda_{T}=\begin{array}{c} School\;performance\\ Self-control\\ Grit \end{array}\left[\begin{array}{c} t_{1}\\ t_{2}\\ t_{3} \end{array}\right]$$

Here the teacher rater effect is treated as a random variable giving rise to the variances *t*_1_^2^, *t*_2_^2^, and *t*_3_^2^, and covariances among the phenotypes (t_1_*t_2_, t_1_*t_3_, t_2_*t_3_). We included SES and sex as fixed covariates, so the expected MZ and DZ covariance matrices conditional on sex and SES are:

$$\begin{array}{c} \begin{array}{c} \;\;\begin{array}{cc} MZ\;twin\;1 & \;\;MZ\;twin\;2 \end{array}\\ \Sigma_{MZ}=\begin{array}{c} MZ\;twin\;1\\ MZ\;twin\;2 \end{array}\left[\begin{array}{cc} \Sigma_{A}+\Sigma_{D}+\Sigma_{E}+\Sigma_{T}\; & \Sigma_{A}+\Sigma_{D}+T*\Sigma_{T}\\ \Sigma_{A}+\Sigma_{D}+T*\Sigma_{T}\; & \Sigma_{A}+\Sigma_{D}+\Sigma_{E}+\Sigma_{T} \end{array}\right] \end{array} \end{array}$$

and

$$\begin{array}{c} \begin{array}{c} \;\;\begin{array}{cc} DZ\;twin\;1 & \;DZ\;twin\;2 \end{array}\\ \Sigma_{DZ}=\begin{array}{c} DZ\;twin\;1\\ DZ\;twin\;2 \end{array}\left[\begin{array}{cc} \Sigma_{A}+\Sigma_{D}+\Sigma_{E}+\Sigma_{T}\; & {\frac{1}{2}}_{A}+\frac{1}{4}*\Sigma_{D}+T*\Sigma_{T}\\ {\frac{1}{2}}_{A}+\frac{1}{4}*\Sigma_{D}+T*\Sigma_{T}\; & \Sigma_{A}+\Sigma_{D}+\Sigma_{E}+\Sigma_{T} \end{array}\right] \end{array} \end{array}$$

The fixed parameter T (coded 0 or 1) in T*Σ_T_ indicates whether the twins share the teacher (*T* = 1) or not (*T* = 0).

Third, we carried out the regression analysis at the level of the genetic and environmental covariance matrices to obtain which of the non‐cognitive factors, self‐control, grit, or their covariance, was the better predictor of school performance. We included the regression of school performance on self‐control and grit at the level of the total genetic Σ_G,_ where Σ_G_ equals Σ_*A*_ + Σ_*D*_ and the environmental covariance matrix Σ_E_. The decomposition of the genetic variance, conditional on sex, SES, and teacher sharing, is

$${{\Sigma_{G}}^{2}}_{\;\_school\;performance|sex,SES,teacher}={b_{G\_SC}}^{2}*{\Sigma_{G\_SC}}^{2}+{b_{G\_grit}}^{2}*{\Sigma_{G\_grit}}^{2}+\left(2*b_{G\_SC}*b_{G\_grit}*\Sigma_{G\_SC,grit}\right)+{{\Sigma_{G}}^{2}}_{\varepsilon }$$

where Σ_G_^2^_ε_ is the genetic prediction error variance and b_G_SC_ and b_G_grit_ are the genetic regression coefficients. The decomposition of environmental variance is

$${{\Sigma_{E}}^{2}}_{\;\_school\;performance|sex,SES,teacher}={b_{E\_SC}}^{2}*{\Sigma_{E\_SC}}^{2}+{b_{E\_grit}}^{2}*{\Sigma_{E\_grit}}^{2}+\left(2*b_{E\_SC}*b_{E\_grit}*\Sigma_{E\_SC,grit}\right)+{{\Sigma_{E}}^{2}}_{\varepsilon }$$

where Σ_E_^2^_ε_ is the environmental prediction error variance and *b*_E_SC_ and *b*_E_grit_ are the environmental regression coefficients.

Statistical analyses were conducted using the OpenMx library (Neale et al., 2016) in R using full information maximum likelihood estimation. We fitted the full model with parameters accommodating the teacher‐rater effect (i.e., the parameters in Λ_T_) estimated freely.

So, in summary, we first fitted a regression to the phenotypic data and then we fitted a regression on the (A + D) and E covariance matrices. The left side of Figure 1 represents the phenotypic regression model, in which self‐control and grit predict school performance. In this model the *R* ^2^, the proportion of phenotypic school performance variance explained is decomposed into three parts: a part directly due to self‐control, a part directly due to grit, and a part due to self‐control and grit together. The third part involves the covariance of self‐control and grit and therefore cannot be attributed to self‐control or grit exclusively.

The right side of Figure 1 presents the (A + D), E regression model, in which we specify the regression relationship at the level of the total genetic covariance matrix (A + D) (comprising the additive genetic and dominance covariance). First, we calculated the *R* ^2^ of the (A + D) variance of school performance, the *R* ^2^ of the E variance of school performance. Second, we calculated the decomposition of the phenotypic school performance variance based on the A + D results and on the E results. Here we expressed the *R* ^2^ of the phenotype school performance in terms of the *R* ^2^ (with three components: a part directly due to grit, a part directly due to self‐control and a part due to their covariance) based on the A + D regression and the *R* ^2^ (again with the same three components) based on the E regression. This allowed us to determine the contribution of A and D, on the one hand, and E, on the other hand, to the *R* ^2^ obtained in the phenotypic regression analyses (Figure 1, right‐hand side).

## RESULTS

### Descriptive statistics

The descriptive statistics (of the raw data) are given in Table 2. School performance correlated about equally high with our non‐cognitive measures (0.40 with self‐control and 0.53 with grit) as with our cognitive measure (0.51 with IQ). Self‐control and grit correlated 0.63.

For the measure of grit, we tested whether a version with just items 1 and 3 shows similar correlations with the other constructs compared to our full measure of grit. We did so, as items 1 and 3 (see Table 1) are conceptually better measures of grit than item 2. As shown in Table 2, our full measure of grit and the items‐1‐and‐3 measure show highly similar correlations with the other construct. We continued in the following analyses with our full measure of grit to maximize the sample size.

### IQ

The assessment of full‐scale IQ in a subsample of 421 children allowed us to investigate if our measures of self‐control and grit were associated with school performance independent of IQ. We tested if self‐control and grit still predict school performance after regressing out IQ. Results indicated grit, but not self‐control, still predicts school performance (*β*_grit_ = 0.27 [S.E. = 0.04] and (*β*_self‐control_ = 0.03 [S.E. = 0.05]). Thus, grit indeed predicts school performance above and beyond the prediction of IQ.

### SES

**FIGURE 4:**
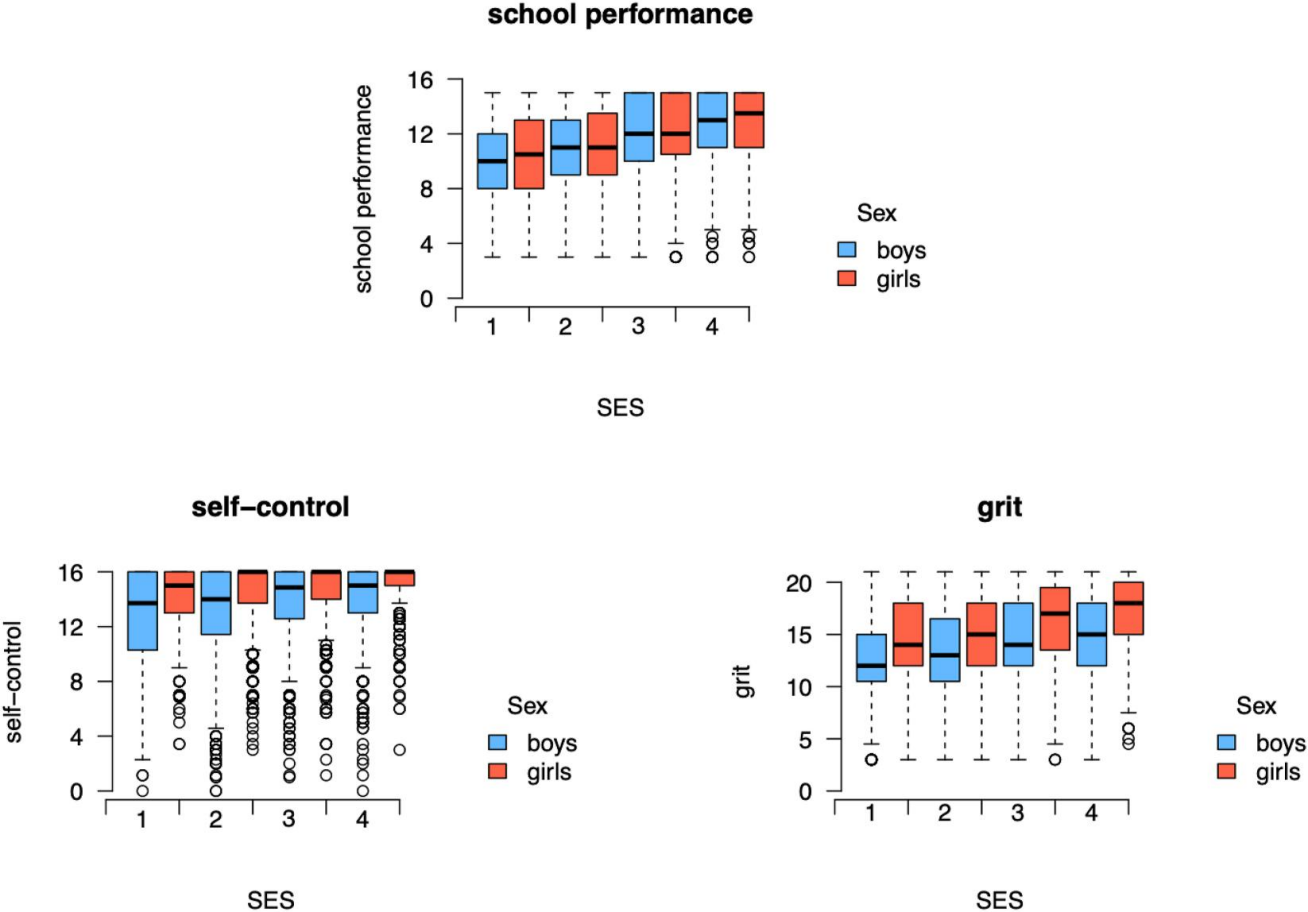
Boxplots of the school performance, self‐control and grit scores separately for boys and girls, and for children from different socio‐economic strata (SES). SES had an effect on the means of school performance, self‐control and grit, but the correlational structure did not differ across SES. We included sex and SES as fixed effects in our model.

Figure 4 displays the means of school performance, self‐control, and grit for boys and girls by SES. The mean school performance, self‐control, and grit vary with SES, with children with higher SES, scoring, on average, higher on school performance, self‐control, and grit. The twin correlations among these variables were highly similar across levels of SES (see Table 3 for correlational structure by SES). The main effects of sex, SES, and sharing the same teacher are displayed in Table 4.

**TABLE 3:** Twin correlations of the raw data (uncorrected for sex, teacher sharing and censoring) for self‐control, grit, and school performance, by SES and zygosity.

Zygosity	SES	Self‐control	Grit	School performance
MZ	Lowest SES	0.66	0.67	0.78
	Lower SES	0.68	0.67	0.72
	Higher SES	0.68	0.71	0.72
	Highest SES	0.63	0.64	0.73
DZ	Lowest SES	0.17	0.15	0.31
	Lower SES	0.22	0.24	0.35
	Higher SES	0.21	0.23	0.30
	Highest SES	0.20	0.22	0.37

All correlations are significant at *p* ≤ .01.

**TABLE 4:** Parameter estimates of the effects of sex, SES and teacher sharing corrected for censoring in the saturated model, with the standard errors (se) between brackets.

	Self‐control	Grit	School performance
*B*_0_ (se)	12.05 (0.05)	11.79 (0.02)	8.85 (0.06)
*b*_sex_ (se)	4.13 (0.11)	4.46 (0.11)	2.76 (0.10)
*b*_SES_ (se)	0.66 (0.02)	0.92 (0.02)	1.07 (0.03)
*b*_teacher_ fixed (se)	0.75 (0.09)	0.55 (0.08)	0.62 (0.10)
Teacher random (se)	0.23 (0.17)	2.11 (0.09)	0.91 (0.03)

*Note*: The teacher random effects equal the parameters in Λ_T_, where *Σ*_T_ = Λ_T_Λ_T_^t^. These parameters squared equal the variance due to the teacher rater effect (i.e., 0.030, 4.026, and 0.928).

### Twin correlations

The twin correlations in Figure 3 suggest the presence of additive genetic effects on school performance (i.e., *r*_MZ_ ≈ 2 * r_DZ_) and additive genetic and as well as dominance effects for self‐control and grit (i.e., *r*_MZ_ > 2 * r_DZ_). Common environmental effects, which are suggested by *r*_MZ_ < 2 * *r*_DZ_, appear to be absent.

### Phenotypic regression model

In the phenotypic model, the regression coefficients equal 0.191 (for self‐control; 95%CIs: 0.131–0.251) and 0.328 (for grit; 95%CIs: 0.252–0.412). Self‐control and grit account for 28.3% of the variance in school performance (conditional on sex, SES and teacher sharing and corrected for censoring). Of this 28.3%, self‐control explained 4.4%, grit explained 13.0%, with the rest, that is, 10.9% due to covariance between self‐control and grit. So, most of the explained variance in school performance is due to grit (46%, i.e., 13.0%/28.3%) and the covariance between self‐control and grit (39%, i.e., 10.9%/28.3%), while self‐control accounted for 16% (i.e., 4.4%/28.3%) of the explained variance in school performance.

### Genetic‐and‐environmental regression model

Subsequently, we fitted the ADE model. In Table 5, the variance‐covariance matrices are presented, with standardized variance components, based on fitting the ADE model. The standardized variance components, corrected for sex and SES, are as follows. The standardized broad‐sense genetic variances (attributable to additive genetic and dominance effect) equal 73.5 (school performance), 68.7% (self‐control), and 57.8% (grit); the standardized unshared environmental variances equal 19.2% (school performance), 31.1% (self‐control), and 20.9% (grit), and the standardized teacher rater variances equal 7.3%, 0.2%, and 21.4%. Conditional on sex, SES, and teacher rater, the standardized broad‐sense genetic variance components are 79% (school performance), 69% (self‐control), and 73% (grit), and the standardized unshared environmental variances are 21% (school performance), 31% (self‐control), and 27% (grit).

**TABLE 5:** Results of fitting the ADE model.

ΣA			ΣD			ΣA + ΣD			ΣE			ΣT		
SP	SC	Grit	SP	SC	Grit	SP	SC	Grit	SP	SC	Grit	SP	SC	Grit
9.01	4.81	4.87	0.29	0.59	1.22	9.30	5.40	6.09	2.43	0.81	0.53	0.93	0.17	1.93
4.81	6.00	2.85	0.59	4.05	5.45	5.40	10.06	8.30	0.81	4.56	2.26	0.17	0.03	0.35
4.87	2.85	2.65	1.22	5.45	8.24	6.09	8.30	10.89	0.53	2.26	3.94	1.93	0.35	4.03

Standardized variances														
*a* ^2^			*d* ^2^			*a* ^2^ + *d* ^2^			*e* ^2^			*t* ^2^		
0.712	0.410	0.141	0.023	0.277	0.437	0.735	0.687	0.578	0.192	0.311	0.209	0.073	0.002	0.214

*Note*: Variance‐covariance matrices conditional on sex and SES and corrected for censoring, and the standardized variance components attributable to additive genetic effects (A), dominance effects (D), unshared environmental effects (E), and the random teacher effect (T). The coefficient *a* ^2^ is the narrow sense heritability. The sum *a* ^2^ + *d* ^2^ is the broad‐sense heritability. The bottom row presents the standardized proportions of variances for each of the traits explained by genetic, dominance, nonshared environmental, and teacher effects. These effects per trait add up to 1 (i.e., a^2^ + d^2^ e^2^ + *t* ^2^ = 1).

In Table 6, we present the explained variance of school performance at the level of A + D and E variance in the top part, and at the level of the phenotypic variance in the bottom part. The results in Table 6 are corrected for sex, SES, and teacher rater effects. Considering the regression as specified at the level of A + D, we found that 38% of the A + D variance of school performance is explained by the genetic (A + D) components of self‐control and grit. The contributions of these genetic components are 4.3% (self‐control), 19.3% (grit) and 14.5% (due to the genetic covariance of self‐control and grit). Considering the regression as specified at the level of E, we found that only 6% of the E variance of school performance is explained by the unshared environmental (E) components of self‐control and grit. The contributions of these unshared environmental components are 4.5% (self‐control), 0.3% (grit) and 1.3% (due to the environmental covariance of self‐control and grit). Of greater interest are the contributions to the phenotypic variance of school performance. Specifically, we know from the phenotypic regression analyses, that self‐control and grit explain about 28.3% of the phenotypic variance of school performance. In the present regression model, we explained slightly more variance, that is, 31.6%. But of this 31.6%, 30.3% is explained by the genetic components of self‐control and grit, and 1.3% is explained by the environmental components of self‐control and grit. The 30.3% breaks down as follows: 3.4% (genetic component of self‐control), 15.3% (genetic component of grit), and 11.5% (genetic covariance of self‐control and grit). The 1.3% breaks down as follows 0.9%, 0.07%, 0.27%. An important finding is therefore that the phenotypic regression analysis is largely a reflection of genetic influences.

**TABLE 6:** School performance variance explained by genetic and environmental components of self‐control and grit, conditional on SES and sex and teacher sharing, and corrected for censoring. The explained variance of school performance at the level of A + D and E variance are presented in the top part, and at the level of the phenotypic variance in the bottom part. Results from the combined genetic covariance structure modeling and regression analysis. Confidence intervals (95%; 95CIs) are displayed below the estimates.

Predictors	SC contribution to *R* ^2^	Grit contribution to *R* ^2^	Cov(SC, grit) contribution to *R* ^2^	Proportion of explained variance *R* ^2^
	At the level of A + D and E variance			
A + D	*b*_G_SC_^2^*s_G_SC_^2^/*s*_G_SP_^2^ = 0.043	*b*_G_grit_^2^*s_G_grit_^2^/*s*_G_SP_^2^ = 0.193	(2*b_G_SC_*s_G___grit_*s_G_SC,grit_)/*s*_G_SP_^2^ = 0.145	*R* ^2^ = 0.38 (38% of A + D variance, s_G_SP_^2^)
E	*b*_E_SC_^2^*s_E_SC_^2^/*s*_E_SP_^2^ = 0.045	*b*_E_grit_^2^*s_E_grit_^2^/*s*_E_SP_^2^ = 0.003	(2*b_E_SC_*s_E___grit_*s_E_SC,grit_)/*s*_E_SP_^2^ = 0.013	*R* ^2^ = 0.06 (6% of E variance, s_E_SP_^2^)
	At the level of the phenotypic variance			
A + D	*b*_G_SC_^2^*s_G_SC_^2^/*s*__SP_^2^ = 0.034	*b*_G_grit_^2^*s_G_grit_^2^/*s*__SP_^2^ = 0.153	2*b_G_SC_*s_G___grit_*s_G_SC,grit_/*s*__SP_^2^ = 0.115	*R* ^2^ = 0.303 (30.3% of phenotypic variance, s__SP_^2^)
95CIs	[0.030–0.067]	[0.081–0.213]	[0.072–0.121]	
E	*b*_E_SC_^2^*s_E_SC_^2^/*s*__SP_^2^ = 0.0094	*b*_E_grit_^2^*s_E_grit_^2^/*s*__SP_^2^ = 0.0007	2*b_E_SC_*s_E___grit_*s_E_SC,grit_/*s*__SP_^2^ = 0.0027	*R* ^2^ = 0.013 (1.3% of phenotypic variance, s__SP_^2^)
95CIs	[0.003–0.011]	[0.0003–0.0009]	[−0.0005–0.0031]	

**FIGURE 5:**
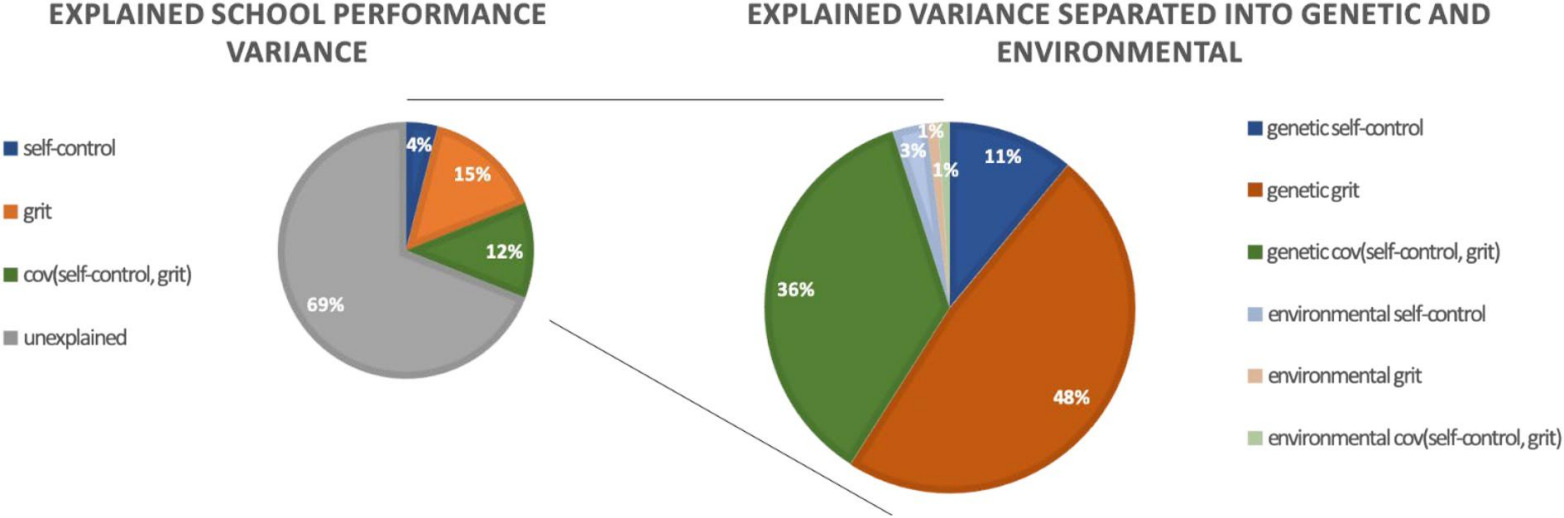
Explained variance in school performance in the genetically informed regression model. Left side: percentages of explained variance in school performance. Right side: percentages of explained variance in school performance by environmental and genetic components of self‐control, grit, and their covariance.

Figure 5 displays the proportions of phenotypic variance in school performance attributable to self‐control, grit, and their covariance (conditional on sex, SES, and teacher sharing and corrected for censoring) based on the genetic covariance structure modeling. The genetic and environmental components of self‐control and grit combined explained 31.6% of the variance in school performance, standardized by the total phenotypic variance. Based on the combined genetic covariance structure modeling and regression analyses (Table 5 and the right part of Figure 5), we conclude that the best predictor of school performance was the genetic (A + D) component of grit. The genetic component of grit accounted for 48.4% (i.e. 15.3%/31.6%) of the total explained phenotypic variance in school performance, and the remaining part is mostly attributable to the genetic covariance between self‐control and grit (36.4%, i.e. 11.5%/31.6%). The environmental components of self‐control, grit, and the covariance between self‐control and grit accounted for only 1.3% of the phenotypic variance. So, this is about 4.1% (i.e., 1.3%/31.6%) of the explained phenotypic variance.

## DISCUSSION

We found that self‐control and grit explained 28.3% of the variance in school performance in the phenotypic model and 31.6% of the variance in school performance in the genetically informed model in 12‐year‐olds. Most of this 31.6% was attributable to the genetic component of grit. Because we employed twin data, we were able to use genetically‐informed regression analyses to disentangle genetic and environmental contributions to the phenotypic associations. Most of the explained variance in school performance by these non‐cognitive factors was accounted for by the genetic components. The best predictor of individual differences in school performance was the genetic component (A + D) of grit. About half of the 31.6% explained variance in school performance was explained by the genetic component of grit, and the remaining half was mostly explained by the genetic component of self‐control and the genetic covariance between self‐control and grit. A very small portion (1.3%) of individual differences in school performance was explained by unique environmental factors, mostly the environmental component of self‐control.

We replicated the finding that self‐control and grit are substantially heritable, with heritability estimates, conditional on sex and SES, of 0.69 and 0.58, respectively. Consistent with the results of other studies, we replicated the absence of common environmental influences (Rimfeld et al., 2016; Willems et al., 2019). The estimate of heritability of grit, conditional on SES and sex in our sample (heritability = 0.58) was somewhat higher than the heritability reported in a previous British study on grit (heritability = ∼0.4, Rimfeld et al., 2016), but the same as a previous Japanese study on grit (heritability = 0.59, Takahashi et al., 2021). The estimate of heritability for school performance (heritability = 0.735) resembled that reported in other studies (Bartels et al., 2002; Pokropek & Sikora, 2015). Based on the twin correlations, we saw no evidence common environmental (C) influence on school performance. However, it is important to consider that we accounted for SES in our model. After correcting for SES, we found that the individual differences in the phenotypes are mainly due to genetic differences. In earlier Dutch studies that did not account for SES, C was also small (<0.10) or absent (de Zeeuw et al., 2016; van Bergen et al., 2018).

Because some twins are in the same class, and shared a teacher, we modeled a random teacher‐rater effect. A noteworthy finding is that sharing a class and thus being rated by the same teacher explained more variance in grit (21.4%) than in self‐control (<1%) or school performance (7.3%). We hypothesized that this may be due to grit, more than self‐control, being influenced by the academic climate in the classroom (Lamb et al., 2012). An Australian study that modeled the classroom effect on achievement test scores found that the variance explained by the classroom effect was only 2%–3% (Grasby et al., 2020). This estimate is based on test scores, so free of a rater effect. Hence, Grasby et al.’s study suggests that the effect of the teacher and other classroom effects on school performance are small. We speculate that our effect is larger, because it includes the rater effect.

Genetic factors contribute strongly to the phenotypic correlations of non‐cognitive skills. Takahashi et al. (2021) identified self‐control and grit, along with conscientious and effortful control, as being part of a conscientious‐related common factor. The four non‐cognitive skills were strongly correlated genetically: the latent common non‐cognitive factor explained 84% of the genetic variance (Takahashi et al., 2021). So, this shows that non‐cognitive factors partly overlap phenotypically, mostly for genetic reasons. The current study indicates that self‐control and grit have distinct aspects; they differ in their contribution to the prediction of school performance. Here, we mostly measured the perseverance aspect of grit, which is the aspect of grit found to be most related to academic outcomes in previous studies (Muenks et al., 2017; Rimfeld et al., 2016).

In a subsample we showed that school performance is similarly correlated with our non‐cognitive measures as with our cognitive measure (IQ; see Table 2). Moreover, grit predicted school performance above and beyond the prediction of IQ. The finding that non‐cognitive factors explain school performance over and above cognitive factors is in line with recent work at the level of measured DNA. Demange et al. (2021) operationalized a general “non‐cognitive factor” by identifying genetic variants (in a genome‐wide association study [GWAS] approach) that are associated with educational attainment, but not with cognition (Demange et al., 2021). Both the non‐cognitive and the cognitive genetic factors predicted socioeconomic success.

A strength of the current study is its large sample of twins, which enabled us to predict children's school performance through self‐control and grit at both the phenotypic, and the genetic and environmental levels. We incorporated the effects of SES, sex, and sharing the same teacher. Another strength of our study is that we corrected for censoring. Our teacher‐rated measures, especially self‐control, showed ceiling effects, meaning that many children scored the highest possible score.

All three main constructs were based on reports from the teacher. For validation and context, we presented in a subsample data based on individual tests (CITO school performance and WISC‐R IQ). Teachers may rate children with better academic achievement as having more self‐control and being grittier, due to response bias or confirmation bias. This hypothesis fits with our observation that we find larger associations between school performance and non‐cognitive skills than previously reported (meta‐analyzed by Credé et al., 2017). We validated the teacher ratings of school performance: The teacher ratings correlated 0.75 with scores on a nationwide standardized educational‐achievement test (i.e., CITO scores). In addition, the heritability estimate of teacher‐rated school performance (heritability = 0.74; Table 5) was the same as that of the CITO scores (heritability = 0.74; de Zeeuw et al., 2016), though the CITO were to a small degree influenced by the shared environment (*c* ^2^ = 0.08; de Zeeuw et al., 2016).

A limitation of the present study concerns the measure of grit. Our measure of grit, emphasizing the perseverance of effort aspect, is weaker than the classical and validated measure, which includes items like “I finish whatever I begin” and “I am diligent” (Duckworth & Quinn, 2009). Our second item (see Table 1) theoretically seems less well related to the grit concept; however, it correlated well with the other two items. A grit measure leaving this item out correlated similarly to self‐control and school performance (Table 2), thus reassuring that our findings are not driven by item 2. Our third item was missing for just over half the sample, but still leaving *N* ∼ 3900.

Our research question concerned prediction, not causation. Accordingly, we used prediction models in cross‐sectional data rather than causal models (Larsson, 2021). Our findings are consistent, but do not prove, a causal effect of non‐cognitive skills on school performance. Alternative explanations of the association are reverse causality (i.e., school performance influences non‐cognitive factors), or a common underlying factor that influences both, without a causal association between non‐cognitive factors and school performance. Future research should tackle these important but challenging research questions.

Our findings concern the status quo: we focused on (the sources of) individual differences, as they exist in the natural situation. That is, we focused on the “what is”, not on the “what could be” as a consequence of intervention (van Bergen et al., 2018). Finding that individual differences in school performance can to a large extent be predicted by the genetic components of self‐control and grit does not mean that these skills are immutable, but reflects that children who are performing well in school oftentimes also are genetically predisposed to be grittier and to have more self‐control. In popular science, cognitive skills like IQ are sometimes thought of as innate talents that are difficult to change, while non‐cognitive skills are thought of as malleable skills that can be nurtured and taught to students (Chang, 2014; Martinez et al., 2022; Sokolowski & Ansari, 2018). Although findings from our and other heritability studies do not speak to trainability, they do show that cognitive skills and non‐cognitive skills are both substantial and similarly heritable, refuting this popular distinction. The potential malleability and trainability of non‐cognitive skills have been investigated with interventions (Sisk et al., 2018). For cognitive skills, Zijlstra, van Bergen, Regtvoort, de Jong, and van der Leij (2021) showed that their 2‐year reading intervention was equally effective in children with and without a family risk for reading difficulties, though the family‐risk group needed more intervention sessions. These findings suggest that a prolonged and tailored intervention can improve children's academic skills, also in those with a genetic predisposition for learning difficulties. Regarding non‐cognitive skills, future work could investigate whether interventions targeting non‐cognitive skills are equally effective in children with and without (a genetic predisposition for) learning difficulties. From our current study, we conclude that whether children do well in school can be predicted by (genetic) components of self‐control and more importantly grit.

## AUTHOR CONTRIBUTIONS

**Sofieke T. Kevenaar**: Conceptualization, Data curation, Formal analysis, Investigation, Methodology, Project administration, Visualization, Writing – original draft. **Conor C. Dolan**: Conceptualization, Formal analysis, Investigation, Methodology, Supervision, Writing – review & editing. **Dorret I. Boomsma**: Conceptualization, Investigation, Methodology, Supervision, Writing – review & editing. **Elsje van Bergen**: Conceptualization, Investigation, Methodology, Supervision, Writing – review & editing.

## CONFLICT OF INTEREST STATEMENT

The authors have declared they have no competing or potential conflicts of interest.

## ETHICAL CONSIDERATIONS

The data collection procedure was ethically approved by the Vaste Commissie Wetenschap en Ethiek (VCWE) at Vrije Universiteit Amsterdam (VCWE‐2021‐111).

## Data Availability

The Netherlands Twin Register (NTR) participants did not agree for their data to be shared publicly. Qualified researchers can request access to NTR data via the procedures outlined on the following website: https://ntr‐data‐request.psy.vu.nl/.
